# New advances in cancer therapy targeting TGF-β signaling pathways

**DOI:** 10.1016/j.omto.2023.100755

**Published:** 2023-12-03

**Authors:** Minyue Shou, Hanyu Zhou, Ling Ma

**Affiliations:** 1 Department of Oncology, The First Affiliated Hospital of Nanjing Medical University, No. 300 Guangzhou Road, Nanjing, China; 2 Department of Oncology, Gusu School, Suzhou Municipal Hospital, The Affiliated Suzhou Hospital of Nanjing Medical University, Suzhou, China; 3 Jiangsu Key Laboratory for Design and Manufacture of Micro-Nano Biomedical Instruments, Southeast University, Nanjing, China

Secretion of transforming growth factor β (TGF-β) is a prominent feature of cancer development and a striking way to inhibit anti-tumor immunity. Elimination effects of TGF-β have far-reaching positive implications for future immunotherapy. In their article, Petersen et al. designed hemagglutinin (HA)-tagged decoy receptors comprised of the extracellular domain of TGF-βRII (TβRII-ecto) attached to glycosylphosphatidylinositol (GPI) anchors, which resulted in a profound inhibition of TGF-β-induced SMAD phosphorylation.^1^ T cells transduced with lentivirus expressing the GPI-ecto-TβRII construct continue to express high levels of interferon γ (IFNγ) and granulocyte-macrophage colony-stimulating factor (GM-CSF), among other cytokines, and efficiently capture and inactivate TGF-β from their environment. It has been shown that GPI-ecto-TβRII-expressing cytotoxic T cells (CTLs) are a modulator of tumor immunosuppression and may reduce the detrimental effects of TGF-β in cancer immunotherapy.

## TGF-β mediates immunosuppression

Immunosuppression remains a major obstacle to the efficacy of therapeutic interventions that can be mediated by multiple mechanisms, including immunoregulatory cytokines.^2^ TGF-β released from various cells in the tumor microenvironment (TME) suppresses anti-tumor activities of immune cells, thus generating an immunosuppressive environment that prevents or attenuates the efficacy of cancer immunotherapies.^3^ Numerous clinical reports associate high TGF-β levels with poor prognosis in different cancer types.^4^ Nonetheless, the development of TGF-β-targeted therapies is proceeding slowly, probably because of the following reasons: (1) the dual mechanism of action of TGF-β in cancer requires further investigation; (2) severe toxicities, such as nervous disorders and cytokine release syndrome (CRS), could also occur as a result of blocking TGF-β activities on normal cells outside of the TME^5^; and (3) the study on the downstream mechanism of TGF-β signaling pathways, biomarkers suitable for patients treated with TGF-β-targeted therapy, and optimal dosing regimens is still in the initial phase. Although T cells expressing the GPI-ecto-TβRII construct did not affect the proliferation or phenotype of transduced T cells in standard culture conditions, further *in vivo* experimental data and preclinical models validating the construct’s safety are still needed.

## Therapeutic approaches to inhibit TGF-β signaling pathway

A range of therapeutic approaches targeting the TGF-β/SMAD signaling pathway and cellular targeting approaches to inhibit TGF-β signaling have been developed, some of which have been used in clinical trials (Table 1). Methods to inhibit TGF-β signaling fall into five main categories.^3^ One class consists of small-molecule inhibitors of TGF-β receptor kinases. These prevent ATP binding to the ATP-binding pocket of the TGF-β receptor (primarily TβRI), thereby blocking SMAD2 and SMAD3 activation. However, despite the simplicity of oral administration, these inhibitors have poor pharmacokinetics and pharmacodynamics. In addition, they are equally effective at inhibiting the receptors for several other TGF-β-related proteins and other kinases. Another class of therapies includes monoclonal antibodies that block TGF-β receptor binding ligands.^6^ Although neutralizing TGF-β antibodies have fine ligand specificity, they are required to effectively interfere with the very high binding affinity of TGF-β to cell surface receptor complexes. Antibodies are expensive to manufacture and may be distributed within tumors inefficiently while having superior pharmacokinetic properties. Other inhibitors have been designed as soluble, high-affinity ligand traps to prevent TGF-β from binding to its receptors. These include Fc-stabilized dimers of the extracellular structural domain of TβRII designed to sequester TGF-β1 and TGF-β3, but not TGF-β2, thus preventing them from binding to transmembrane TβRII receptors. The HA-tagged decoy receptors constructed by Petersen et al. fall into this category. In addition, some antibodies and small molecules target the TGF-β activation process and thus provide selective cell- or tissue-type inhibition of TGF-β signaling. Finally, antisense oligonucleotides (ASOs) are single-stranded DNA or RNA sequences composed of 15–25 nucleotides that directly inhibit the synthesis of TGF-β by targeting and interfering with the transcription or translation process. Due to their high specificity, efficiency, safety, and low toxicity, ASOs have shown broad application prospects in gene therapy. However, development in clinical practice faces many limitations, such as the instability of ASO molecules and insufficient uptake by cells.^7^

**Table 1:** Therapeutic approaches to inhibit TGF-β signaling pathway

Drugs and targets	Research status	Main adverse events	Tumor species	NCT no.
**Small-molecule inhibitors that inhibit TGF-βR kinase activity**				

Vactosertib (FEW-7197): TGF-βR1/ALK5 kinase activity inhibitor	vactosertib had an acceptable tolerability and safety profile	N/D	NSCLC	NCT03732274 (unknown status)
				NCT04515979 (recruiting)
			CRC, GC	NCT03724851 (unknown status)
				NCT04893252 (recruiting)
				NCT05400122 (recruiting)
				NCT03844750 (recruiting)
			PCA	NCT04258072 (recruiting)
			solid tumors	NCT02160106 (complete)
Galunisertib (LY21557299): TGF-βR1 kinase inhibitor	galunisertib had an acceptable tolerability and safety profile; galunisertib prolonged overall survival (OS) in HCC and PCA while failing to demonstrate improved OS in malignant glioma	fatigue, nausea, vomiting, decreased platelet count, neutropenia, thrombocytopenia, impaired hearing, impaired liver function, palmar-plantar erythrodysesthesia syndrome, pneumonia, and respiratory tract infection	HCC	NCT02240433 (complete)
				NCT01246986 (complete)
				NCT02178358 (complete)
				NCT02906397 (complete)
			PCA	NCT02734160 (complete)
				NCT01373164 (complete)
				NCT02154646 (complete)
			glioma	NCT01220271 (complete)
				NCT01582269 (preliminarily complete)
			BC, UCS, OC	NCT03206177 (preliminarily complete)
				NCT02672475 (active)
				NCT02538471 (terminated)
			CRC	NCT02688712 (active)
			prostate cancer	NCT02452008 (recruiting)
			solid tumors	NCT02423343 (complete)
				NCT02304419 (complete)
				NCT01722825 (complete)
LY3200882: TGF-βR1 inhibitor	LY3200882 was safe and well tolerated with preliminary anti-tumor activity	headache, fatigue, and increased alanine aminotransferase	CRC	NCT04031872 (unknown status)
			solid tumors	NCT02937272 (preliminarily complete)
PF06952229: TGF-βR1 inhibitor	N/D	N/D	solid tumors	NCT03685591 (terminated)

**TGF-β antibodies**				

Fresolimumab (GC1008): human IgG4κ monoclonal TGF-β1,2,3 antibody	fresolimumab demonstrated acceptable safety and preliminary evidence of anti-tumor activity, and the higher dose had a favorable systemic immune response and experienced longer median OS	fatigue, anorexia, cough, and the development of reversible cutaneous keratoacanthomas/squamous cell carcinomas and hyperkeratosis	NSCLC	NCT02581787 (complete)
			BC	NCT01401062 (complete)
			brain tumors	NCT01472731 (complete)
			MPM	NCT01112293 (complete)
			RCC, MM	NCT00356460 (complete)
				NCT00923169 (complete)
SAR439459: a pan-TGF-β neutralizing antibody	occurrence of objective responses in multiple indications was not enough to warrant further exploration in view of competitive landscape and some toxicity	bleeding events	solid tumors	NCT03192345 (terminated)
				NCT04729725 (terminated)
SRK-181: anti-latent TGF-β1 monoclonal antibody	N/D	N/D	solid tumors	NCT04291079 (recruiting)
NIS793: human anti-TGF-β1 IgG2 monoclonal antibody	efficacy and safety have not been established	N/D	PCA	NCT04390763 (active)
				NCT04935359 (active)
				NCT05417386 (recruiting)
				NCT05546411 (terminated)
			CRC	NCT04952753 (active)
			solid tumors	NCT02947165 (complete)
IMC-TR1 (LY 3022859): an anti-TGF-βR2 IgG1 monoclonal antibody	the maximum tolerated dose for LY3022859 was not determined	worsening symptoms and uncontrolled cytokine release	solid tumors	NCT01646203 (complete)

**TGF-β ligand traps**				

Bintrafusp alfa (GSK-4045154, M7824, MSB0011359C): a bifunctional fusion protein that blocks programmed death ligand 1 (PD-L1) and neutralizes TGF-β	bintrafusp alfa had a manageable safety profile but may not have good anti-tumor activity in several trials; GlaxoSmithKline decided not to move forward with the development of M7824; bintrafusp alfa had some effectiveness in CC; currently, trials in NSCLC and CC and BC are still ongoing	nausea, asthenia, anemia, thrombocytopenia, decreased lymphocyte count, decreased appetite, pruritus, rash, impaired liver function, constipation, oral hemorrhage, hematuria, diarrhea, skin infection secondary to localized bullous pemphigoid, asymptomatic lipase increase, colitis with associated anemia, and gastroparesis with hypokalemia	UC	NCT04349280 (preliminarily complete)
				NCT04501094 (terminated)
			pleural mesothelioma	NCT05005429 (recruiting)
			BTC	NCT04727541 (terminated)
				NCT03833661 (complete)
				NCT04066491 (complete)
				NCT04708067 (recruiting)
			NPC	NCT04396886 (active)
			HNSCC	NCT04428047 (terminated)
				NCT04247282 (complete)
				NCT04220775 (complete)
			women’s tumors (BC, CC)	NCT04489940 (terminated)
				NCT04246489 (complete)
				NCT03620201 (active)
				NCT04551950 (complete)
				NCT03579472 (terminated)
				NCT03524170 (complete)
				NCT04296942 (terminated)
				NCT04296942 (terminated))
			NSCLC	NCT04396535 (active)
				NCT04971187 (terminated)
				NCT04560686 (terminated)
				NCT03840915 (complete)
				NCT03631706 (active)
				NCT03840902 (terminated)
			ESCC	NCT04481256 (recruiting)
			TC	NCT04417660 (recruiting)
			PCA	NCT04327986 (terminated)
			CRC	NCT03436563 (active)
				NCT04491955 (complete)
			solid tumors	NCT05061823 (active)
				NCT02699515 (complete)
				NCT04235777 (recruiting)
				NCT02517398 (complete)
				NCT04789668 (active)
AVID200: a TGF-β receptor ectodomain-IgG Fc fusion protein inhibitor of TGF-β	N/D	N/D	malignancies	NCT03834662 (preliminarily complete)

**Selective interference with activation of latent TGF-β complexes**				

Livmoniplimab (ABBV-151, ARGX-115): humanized anti-LRRC32 (GARP/TGF-β1) monoclonal antibody	N/D	N/D	HCC	NCT05822752 (recruiting)
			solid tumors	NCT03821935 (recruiting)

**ASOs directly inhibit TGF-β synthesis**				

Trabederson (AP12009, OT101): phosphorothioate antisense oligodeoxynucleotide specific for the mRNA of TGF-β2	intratumorally administered OT101 exhibits clinically meaningful single-agent activity and induces durable CR/PR/SD	headache, pyrexia, and meningitis	glioma	NCT00431561 (complete)
				NCT00761280 (terminated)
			solid tumors	NCT00844064 (complete)
Belagenpanatucel-L (Lucanix): anti-TGF-β tumor vaccine (transfection of TGF-β ASO into cancer cell lines)	belagenpanatucel-L was well tolerated, while the overall trial did not meet its survival endpoint	injection site reaction, pain, breathing problem, cough, fatigue, erythema, and induration	NSCLC	NCT01058785 (complete)
				NCT00676507 (complete)
Vigil (vital): incorporate the rhGMCSF transgene and the bifunctional shRNA furin (to block proprotein conversion to active TGF-β1 and -β2)	vigil was well tolerated but the primary endpoint was not met, but vigil elicited an immune response correlating with prolonged survival and still exhibited clinical benefit in selected gynecological cancers; business decision to pursue other indications	neutropenia, abdominal pain, soft tissue infection, tumor pain, skin ulceration, acute cholecystitis, pharyngitis, intracerebral hemorrhage, edema extremities, and dyspnea	MM	NCT02574533 (complete)
				NCT01453361 (terminated)
			women’s cancers	NCT02725489 (complete)
				NCT03073525 (complete)
				NCT02346747 (active)
				NCT01551745 (complete)
				NCT01867086 (complete)
			Ewing’s sarcoma	NCT03495921 (terminated)
				NCT02511132 (complete)
			CRC	NCT01505166 (terminated)
			solid tumors	NCT01061840 (complete)

BC, breast cancer; BTC, biliary tract cancer; CC, cervical cancer; CRC, colorectal cancer; ESCC, esophageal squamous cell carcinoma; HCC, hepatocellular carcinoma; HNSCC, head and neck squamous cell carcinoma; GC, gastric cancer; MM, malignant melanoma; MPM, malignant pleural mesothelioma; N/D, no data; NPC, nasopharyngeal carcinoma; NSCLC, non-small cell lung cancer; OC, ovarian cancer; PCA, pancreatic cancer; RCC, renal cell carcinoma; TC, thymic carcinoma; UC, urothelial carcinoma; UCS, uterine carcinosarcoma.

## Combining TGF-β blockade with anti-cancer therapies

Inhibition of TGF-β signaling enhances the efficacy of other therapeutic approaches because it suppresses immunosuppressive activity, angiogenic responses, and activation of cancer-associated fibroblast (CAF) populations in the TME. In addition, TGF-β-signaling-driven epithelial-mesenchymal transition (EMT) in cancer cells also promotes cancer drug resistance and cancer stem cell properties. Therefore, targeting EMT that inhibits TGF-β signaling and/or cancer cells in combination with cytotoxicity or radiation therapy is expected to inhibit cancer progression. Due to the current emphasis on immunotherapy, the pathway of combining anti-TGF-β with checkpoint inhibitors is being closely scrutinized. It has been observed that increased TGF-β signaling in the TME is associated with worse overall survival and resistance to immunotherapeutic blockade of PD-L1 or its receptor, PD-1.^8^ Considering that TGF-β, CTLA-4, and PD-L1/PD-1 signaling act as parallel immunosuppressive pathways that inhibit CTL, natural killer (NK) cell, and macrophage activity through different mechanisms, blocking TGF-β signaling should inhibit immunosuppressive activity and improve the success rate of immune checkpoint inhibitors (ICIs) or other immunotherapeutic approaches. Several combination therapies have been evaluated preclinically in mouse cancer models. Therefore, the novel GPI-anchored, dominant-negative TβRII could be further combined with ICIs to evaluate the efficacy and adverse effects in preclinical models for further consideration of clinical applications.

TGF-β inhibition has been pursued to enhance the efficacy of chimeric antigen receptor (CAR)-T cell therapies since TGF-β inhibits cytotoxic T lymphocyte activation. The TβRI inhibitor SM16A enhances T cell metastasis and anti-tumor efficacy in mesothelioma mice.^9^ Further modifications to the CAR-T-mediated approach led to the engineering of T cells expressing CAR with extracellular single-chain variable fragments derived from neutralizing TGF-β antibodies. These CAR-T cells respond to TGF-β by dimerizing the TGF-β-binding CAR, leading to T cell activation. Thus, instead of performing its normal immunosuppressive role, TGF-β acts as an effective immunostimulant for these CAR-T cells.^10^ Such design may enable the targeting of CAR-T cells to areas in the TME with high levels of TGF-β. Petersen et al. mentioned that CAR and GPI-TβRII co-expressing adoptive T cells (ATCs) were able to kill antigen-expressing cancer cell spheroids markedly efficiently.

In summary, the GPI-ecto-TβRII construct designed by Petersen et al. is a promising therapy strategy due to its higher affinity than what has been previously established, T cell activation, and TGF-β depletion from TME, thereby antagonizing tumor immunosuppression. However, the efficacy of this construct has not been compared or combined with ICIs or CAR-T therapies, which cannot reflect superiority in clinical application. Most importantly, although the TME has a great impact on tumor therapeutic efficacy, there is a lack of *in vivo* research results. In addition, drugs that inhibit TGF-β signaling pathways, in the past, have proved to have a variety of adverse effects, so the safety of this therapeutic intervention needs to be further explored. Finally, even with nice results in preclinical models, the failure of multiple drugs that inhibit the TGF-β signaling pathway suggests that more high-level evidence-based recommendations are needed.
